# Exenatide once weekly for the treatment of type 2 diabetes: effectiveness and tolerability in patient subpopulations

**DOI:** 10.1111/j.1742-1241.2012.03006.x

**Published:** 2012-11

**Authors:** R Pencek, A Blickensderfer, Y Li, S C Brunell, S Chen

**Affiliations:** Amylin Pharmaceuticals, Inc.San Diego, CA, USA

## Abstract

**Objective:**

Patient numbers in individual diabetes trials are often too limited to assess the effect of a treatment by different patient characteristics, and meta-analyses often do not include patient-level data. The purpose of this pooled analysis was to evaluate the efficacy and tolerability of exenatide once weekly (EQW) in patients with type 2 diabetes grouped into subpopulations by key demographic characteristics.

**Methods:**

This *post hoc* analysis included data from patients who received EQW in seven randomised, controlled phase 3 trials that were 24–30 weeks in duration. Patients were classified into subpopulations on the basis of their baseline age (< 65 or ≥ 65 years), gender (male or female), race (White, Black, Asian, Hispanic), duration of diabetes (< 10 years, ≥ 10 years) and body mass index (BMI; < 25, ≥ 25 to < 30, ≥ 30 to < 35, ≥ 35 to < 40 or ≥ 40 kg/m^2^).

**Results:**

A total of 1719 patients were included in this analysis of patient subpopulations. All subpopulations experienced significant improvements from baseline in haemoglobin A1C, fasting glucose and body weight. Most subpopulations experienced significant improvements in blood pressure and lipid parameters. Overall, the most common AEs were hypoglycaemia (16.4% overall; 2.3% in patients not on concomitant sulfonylurea), nausea (14.7%), diarrhoea (10.9%) and nasopharyngitis (7.2%).

**Conclusion:**

These results show that the treatment of type 2 diabetes with EQW for 24–30 weeks was associated with significant improvements in glycaemic control and body weight, irrespective of age, gender, race, duration of diabetes or BMI. The most common adverse events were gastrointestinal in nature.

**What’s known**

There have been few pooled analyses of data from studies of glucagon-like peptide-1 receptor agonists that evaluated the efficacy and tolerability of these drugs grouped by different subpopulations of patients. However, such an analysis of data from exenatide twice daily clinical studies found that this medication was effective and tolerated in patients across a range of baseline and demographic characteristics.

**What’s new**

The results of the current analysis revealed that the treatment with exenatide once weekly was associated with significant improvements in glycaemic control and body weight, irrespective of age, gender, race, duration of diabetes or body mass index. There did not appear to be any striking differences in the incidences of adverse events between subpopulations, and the rates of withdrawal because of adverse events was similar between subpopulations.

## Introduction

Two formulations of the glucagon-like peptide-1 receptor agonist (GLP-1RA) exenatide are now available in the USA and the European Union for the improvement of glucose control in patients with type 2 diabetes: exenatide twice daily (BID) and exenatide once weekly (EQW). In the EQW formulation, exenatide is encapsulated in poly(d,l-lactide-co-glycolide) microspheres, allowing for gradual delivery of exenatide (1). With weekly injections, the therapeutic threshold of exenatide is reached 2 weeks after initiation and steady-state concentrations occur within 6–10 weeks (2). Exenatide once weekly is approved in the USA for the treatment of type 2 diabetes as monotherapy or as adjunctive therapy in patients who are treated with metformin (MET), a sulfonylurea (SU), a thiazolidinedione (TZD), a combination of MET and an SU, or a combination of MET and a TZD (3).

Patients with type 2 diabetes can have a wide range of baseline and demographic characteristics. However, data are limited on the effects of these characteristics on the efficacy and tolerability of GLP-1RAs. Both liraglutide and exenatide BID have been reported to be effective in patients of different races (4–6). Results from a pooled analysis of liraglutide clinical studies showed that liraglutide was similarly effective on glycaemic end-points in patients aged < 65 years as in those aged ≥ 65 years (7). Pooled analyses of exenatide BID studies reported data stratified by a variety of baseline characteristics (8,9). In those analyses, exenatide BID was associated with improved glycaemic control and body weight, and beneficial effects on blood pressure and lipids in patients regardless of baseline characteristics.

Numerous controlled clinical trials with EQW have been conducted in patients with a range of baseline and demographic characteristics. However, a comprehensive analysis of the efficacy and tolerability of EQW stratified by these characteristics has not yet been reported. In this pooled analysis, the efficacy and tolerability of EQW in patients stratified into subpopulations [i.e. age, gender, race, body mass index (BMI) and duration of diabetes] were explored.

## Research design and methods

### Study and patient selection

**Figure 1:**
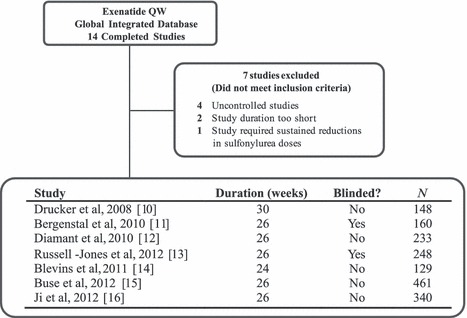
Flowchart of the studies included in the analyses

Fourteen clinical studies with a 2-mg dose of EQW have been conducted. Studies included in this analysis were selected based on the following criteria: (i) randomised and controlled; (ii) 24–30 weeks in duration; (iii) concomitant SU use was managed consistently with the EQW development programme (i.e. studies that required sustained reductions in SU doses were excluded); (iv) end-points included A1C, fasting glucose (FG), body weight, blood pressure and lipids. Seven studies from the phase 3 development programme met these criteria (see Figure 1 for a flow chart of the included/excluded studies). The main results of these studies have been previously reported (10–16). All studies included in this study were conducted in accordance with the Declaration of Helsinki (1964), including the current Seoul revision (2008, when applicable), and were consistent with Good Clinical Practice and applicable laws and regulations. All patients provided written informed consent before participation in the original clinical trials.

### Analysis methods

The following subpopulations were identified as being of clinical importance: age (< 65 or ≥ 65 years), gender (male or female), race [White, Black, Asian (either South or East Asian) or Hispanic], duration of diabetes (< 10 or ≥ 10 years) and BMI (< 25, ≥ 25 to < 30, ≥ 30 to < 35, ≥ 35 to < 40, or ≥ 40 kg/m^2^). Patients were categorised into each of the subpopulations for the analysis. Baseline and demographic characteristics (mean ± SD where applicable) were reported for each subpopulation.

Baseline, end-point and change from baseline of A1C, FG, body weight, blood pressure and lipid parameters were summarised descriptively for all subpopulations. Ninety-five per cent confidence intervals (CIs) were calculated for the change from baseline data. p Values were provided for the within-group comparison for end-point vs. baseline using the paired *t*-test. The last observation carried forward method was implemented to handle missing data from patients who discontinued early. Forest plots were provided for change in A1C, FG and body weight.

Adverse events (AEs) with an incidence of ≥ 5% in the overall population were tabulated. AEs leading to withdrawal were tabulated. Hypoglycaemia was assessed and characterised using the following criteria: major hypoglycaemia included events that (i) in the judgment of the investigator or physician, resulted in a loss of consciousness, seizure, or coma and resolved after the administration of glucagon or glucose; or (ii) required third-party assistance to resolve and had a glucose value of < 3 mmol/l. Minor hypoglycaemia was defined as a report of symptoms consistent with hypoglycaemia and a glucose value of < 3 mmol/l prior to treatment of the episode. SAS 9.2® (Statistical Analysis Software, Cary, NC) was used for all analyses.

## Results

### Baseline and demographic characteristics

A total of 1719 patients were included in the analyses. Only 20 patients were in the ‘Other’ racial category and were excluded from the race subpopulation analysis. Additionally, 11 patients did not have baseline BMI measurements and were excluded from the BMI subpopulation analysis. Overall, the mean (SD) age was 55 (10) years, the mean baseline A1C was 8.5% (1.1%) and the mean baseline weight was 87.4 (20.5) kg. Of note, there were differences in the numbers of patients in the different racial subpopulations, with more White than non-White patients (Table 1). Also, there were relatively few patients in the ≥ 65-year age subpopulation and in the ≥ 10-year duration of diabetes subpopulation, compared with the < 65-year age subpopulation and the < 10-year duration of diabetes subpopulation, respectively (Tables 1 and 2).

**Table 1:** Demographic and baseline characteristics in the age, gender and racial subpopulations

Parameter	Age		Gender		Race			
		
	< 65 years (*N* = 1427)	≥ 65 years (*N* = 292)	Male (*N* = 944)	Female (*N* = 775)	White (*N* = 1000)	Black (*N* = 47)	Asian (*N* = 505)	Hispanic (*N* = 147)
Age, mean (SD), years	52 (8.5)	69 (3.9)	55 (10.2)	55 (10.4)	57 (9.6)	50 (10.4)	53 (10.7)	52 (11.0)
Male, no. (%)	788 (55.2)	156 (53.4)	–	–	564 (56.4)	27 (57.4)	272 (53.9)	72 (49.0)
**Race, no. (%)**								
White	814 (57.0)	186 (63.7)	564 (59.7)	436 (56.3)	–	–	–	–
Black	43 (3.0)	4 (1.4)	27 (2.9)	20 (2.6)	–	–	–	–
Asian	425 (29.8)	80 (27.4)	272 (28.8)	233 (30.1)	–	–	–	–
Hispanic	127 (8.9)	20 (6.8)	72 (7.6)	75 (9.7)	–	–	–	–
Other	18 (1.3)	2 (0.7)	9 (1.0)	11 (1.4)	–	–	–	–
Weight, mean (SD), kg	88.6 (20.8)	81.5 (17.9)	92.8 (20.4)	80.9 (18.6)	95.6 (18.8)	98.9 (19.7)	70.6 (12.5)	85.9 (18.1)
BMI, mean (SD), kg/m^2^	31.5 (5.7)	30.2 (5.4)	30.8 (5.4)	31.9 (6.0)	33.2 (5.3)	33.9 (5.7)	26.8 (3.6)	32.5 (5.1)
A1C, mean (SD), %	8.5 (1.1)	8.2 (0.9)	8.5 (1.1)	8.5 (1.1)	8.3 (1.0)	8.8 (1.36)	8.7 (1.1)	8.7 (1.3)
Fasting glucose, mean (SD), mmol/l	9.5 (2.7)	9.2 (2.4)	9.5 (2.6)	9.5 (2.6)	9.8 (2.6)	10.0 (3.3)	9.0 (2.4)	9.3 (2.7)
Duration of diabetes, mean (SD), years	6.2 (5.0)	9.8 (6.7)	6.8 (5.6)	6.9 (5.4)	6.8 (5.6)	5.4 (4.7)	7.0 (5.3)	7.0 (6.0)
**Background treatment, no. (%)**								
Diet and exercise	176 (12.3)	30 (10.3)	113 (12.0)	93 (12.0)	144 (14.4)	8 (17.0)	36 (7.1)	17 (11.6)
Metformin	582 (40.8)	102 (34.9)	361 (38.2)	323 (41.7)	417 (41.7)	27 (57.4)	128 (25.3)	97 (66.0)
Metformin + sulfonylurea	512 (35.9)	114 (39.0)	349 (37.0)	277 (35.7)	345 (34.5)	7 (14.9)	246 (48.7)	24 (16.3)
Metformin + thiazolidinedione	54 (3.8)	6 (2.1)	39 (4.1)	21 (2.7)	35 (3.5)	1 (2.1)	19 (3.8)	5 (3.4)
Sulfonylurea	39 (2.7)	19 (6.5)	31 (3.3)	27 (3.5)	27 (2.7)	0 (0.0)	31 (6.1)	0 (0.0)
Other	64 (4.5)	21 (7.2)	51 (5.4)	34 (4.4)	32 (3.2)	4 (8.5)	45 (8.9)	4 (2.7)

BMI, body mass index.

**Table 2:** Demographic and baseline characteristics in the duration of diabetes and body mass index subpopulations

Parameter	Duration of diabetes		BMI (kg/m^2^)				
	
	< 10 years (*N* = 1268)	≥ 10 years (*N* = 451)	< 25 (*N* = 216)	≥ 25 to < 30 (*N* = 560)	≥ 30 to < 35 (*N* = 512)	≥ 35 to < 40 (*N* = 266)	≥ 40 (*N* = 154)
Age, mean (SD), years	53 (10.2)	60 (9.0)	56 (10.8)	56 (10.5)	55 (9.9)	54 (10.2)	53 (10.1)
Male, no. (%)	701 (55.3)	243 (53.9)	127 (58.8)	330 (58.9)	283 (55.3)	133 (50.0)	63 (40.9)
**Race, no. (%)**							
White	745 (58.8)	255 (56.5)	33 (15.3)	263 (47.0)	369 (72.1)	205 (77.1)	130 (84.4)
Black	40 (3.2)	7 (1.6)	3 (1.4)	10 (1.8)	14 (2.7)	13 (4.9)	7 (4.5)
Asian	362 (28.5)	143 (31.7)	175 (81.0)	225 (40.2)	86 (16.8)	6 (2.3)	2 (1.3)
Hispanic	107 (8.4)	40 (8.9)	3 (1.4)	55 (9.8)	40 (7.8)	37 (13.9)	12 (7.8)
Other	14 (1.1)	6 (1.3)	2 (0.9)	7 (1.3)	3 (0.6)	5 (1.9)	3 (1.9)
Weight, mean (SD), kg	88.9 (20.3)	83.3 (20.4)	62.4 (8.2)	76.1 (10.8)	91.5 (12.5)	105.2 (14.1)	120.5 (14.9)
BMI, mean (SD), kg/m^2^	31.7 (5.6)	30.1 (5.7)	23.4 (1.3)	27.5 (1.4)	32.3 (1.4)	37.3 (1.4)	42.6 (1.7)
A1C, mean (SD), %	8.4 (1.1)	8.6 (1.1)	8.7 (1.0)	8.5 (1.1)	8.4 (1.1)	8.4 (1.1)	8.5 (1.1)
Fasting glucose, mean (SD), mmol/l	9.4 (2.6)	9.8 (2.7)	9.3 (2.5)	9.3 (2.6)	9.6 (2.5)	9.7 (2.9)	9.9 (2.6)
Duration of diabetes, mean (SD), years	4.2 (2.7)	14.3 (4.6)	8.6 (6.0)	7.1 (5.6)	6.2 (5.2)	6.3 (5.5)	6.5 (5.4)
**Background treatment, no. (%)**							
Diet and exercise	194 (15.3)	12 (2.7)	16 (7.4)	73 (13.0)	65 (12.7)	36 (13.5)	16 (10.4)
Metformin	556 (43.8)	128 (28.4)	52 (24.1)	224 (40.0)	210 (41.0)	129 (48.5)	68 (44.2)
Metformin + sulfonylurea	387 (30.5)	239 (53.0)	101 (46.8)	213 (38.0)	176 (34.4)	79 (29.7)	50 (32.5)
Metformin + thiazolidinedione	38 (3.0)	22 (4.9)	6 (2.8)	12 (2.1)	23 (4.5)	11 (4.1)	7 (4.5)
Sulfonylurea	38 (3.0)	20 (4.4)	20 (9.3)	16 (2.9)	12 (2.3)	4 (1.5)	5 (3.2)
Other	55 (4.3)	30 (6.7)	21 (9.7)	22 (3.9)	26 (5.1)	7 (2.6)	8 (5.2)

BMI, body mass index.

There were also notable differences across the different subpopulations with regard to baseline and demographic characteristics. As may be expected, older patients (≥ 65 years) tended to have a greater duration of diabetes than did younger patients (< 65 years). Body weight was typically lower in the older patients, females and Asians than that in, respectively, younger patients, males and patients in other racial subpopulations. There was higher SU use among White and Asian patients than that among Black and Hispanic patients. Baseline A1C tended to be lower in White patients than that in patients in other racial subpopulations.

### Efficacy

#### Age

**Figure 2:**
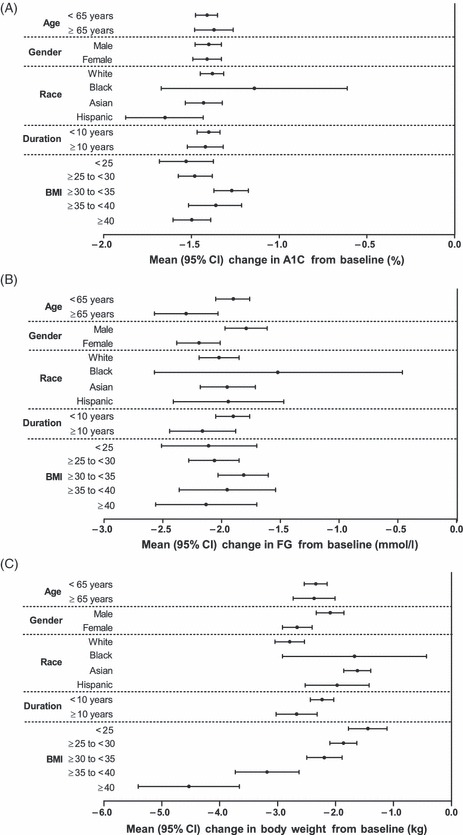
Mean (95% confidence interval) change from baseline to end-point in A1C (A), fasting glucose (B) and body weight (C) for each subpopulation

Significant improvements from baseline in A1C, FG, body weight, blood pressure and lipids (except triglycerides and HDL cholesterol in patients ≥ 65 years) were seen in both younger (< 65 years) and older (≥ 65 years) patients with EQW treatment (Table 3; Figure 2).

**Table 3:** Efficacy end-points in the age, gender and racial subpopulations

Parameter	Age		Gender		Race			
		
	< 65 years (*N* = 1427)	≥ 65 years (*N* = 292)	Male (*N* = 944)	Female (*N* = 775)	White (*N* = 1000)	Black (*N* = 47)	Asian (*N* = 505)	Hispanic (*N* = 147)
**A1C, %**								
Baseline	8.5 (0.03)	8.2 (0.06)	8.5 (0.04)	8.5 (0.04)	8.3 (0.03)	8.8 (0.20)	8.7 (0.05)	8.7 (0.11)
End-point	7.1 (0.03)	6.8 (0.05)	7.1 (0.04)	7.1 (0.04)	7.0 (0.03)	7.6 (0.25)	7.2 (0.05)	7.1 (0.10)
Δ from baseline	−1.4 (−1.5, −1.4)	−1.4 (−1.5, −1.3)	−1.4 (−1.5, −1.3)	−1.4 (−1.5, −1.3)	−1.4 (−1.5, −1.3)	−1.1 (−1.7, −0.6)	−1.4 (−1.5, −1.3)	−1.7 (−1.9, −1.4)
p Value*	< 0.0001	< 0.0001	< 0.0001	< 0.0001	< 0.0001	< 0.0001	< 0.0001	< 0.0001
**FG, mmol/l**								
Baseline	9.54 (0.07)	9.22 (0.14)	9.50 (0.09)	9.48 (0.10)	9.75 (0.08)	9.95 (0.50)	9.01 (0.11)	9.29 (0.23)
End-point	7.62 (0.07)	6.97 (0.11)	7.70 (0.08)	7.28 (0.08)	7.72 (0.08)	8.27 (0.52)	7.05 (0.09)	7.34 (0.21)
Δ from baseline	−1.90 (−2.05, −1.76)	−2.30 (−2.57, −2.03)	−1.79 (−1.97, −1.61)	−2.19 (−2.38, −2.01)	−2.02 (−2.19, −1.85)	−1.52 (−2.57, −0.46)	−1.95 (−2.18, −1.71)	−1.94 (−2.41, −1.47)
p Value*	< 0.0001	< 0.0001	< 0.0001	< 0.0001	< 0.0001	0.0058	< 0.0001	< 0.0001
**Weight, kg**								
Baseline	88.6 (0.55)	81.5 (1.05)	92.8 (0.66)	80.9 (0.67)	95.6 (0.60)	98.9 (2.88)	70.6 (0.56)	85.9 (1.49)
End-point	86.3 (0.54)	79.2 (1.02)	90.6 (0.65)	78.3 (0.66)	92.8 (0.59)	97.0 (2.85)	69.0 (0.56)	84.1 (1.54)
Δ from baseline	−2.3 (−2.5, −2.1)	−2.4 (−2.7, −2.0)	−2.1 (−2.3, −1.9)	−2.7 (−2.9, −2.4)	−2.8 (−3.0, −2.5)	−1.7 (−2.9, −0.4)	−1.6 (−1.9, −1.4)	−2.0 (−2.5, −1.4)
p Value*	< 0.0001	< 0.0001	< 0.0001	< 0.0001	< 0.0001	0.0096	< 0.0001	< 0.0001
**SBP, mmHg**								
Baseline	130 (0.4)	135 (0.8)	131 (0.5)	130 (.05)	132 (0.5)	130 (1.6)	130 (0.7)	125 (1.3)
End-point	127 (0.4)	131 (0.9)	129 (0.5)	126 (0.5)	129 (0.5)	130 (2.0)	126 (0.7)	123 (1.1)
Δ from baseline	−3.0 (−3.7, −2.3)	−3.8 (−5.6, −2.0)	−2.4 (−3.3, −1.5)	−4.0 (−5.0, −3.0)	−2.8 (−3.6, −1.9)	0.3 (−3.6, 4.2)	−4.3 (−5.6, −2.9)	−2.6 (−4.7, −0.5)
p Value*	< 0.0001	< 0.0001	< 0.0001	< 0.0001	< 0.0001	0.8704	< 0.0001	0.0152
**DBP, mmHg**								
Baseline	80 (0.2)	77 (0.5)	80 (0.3)	78 (0.3)	79 (0.3)	81 (1.2)	79 (0.4)	78 (0.8)
End-point	79 (0.2)	76 (0.5)	79 (0.3)	77 (0.3)	79 (0.3)	80 (1.4)	78 (0.4)	77 (0.7)
Δ from baseline	−0.7 (−1.2, −0.3)	−1.3 (−2.4, −0.2)	−0.6 (−1.2, −0.0)	−1.1 (−1.7, −0.5)	−0.6 (−1.2, −0.1)	−1.5 (−4.5, 1.5)	−1.1 (−1.9, −0.3)	−1.0 (−2.2, 0.3)
p Value*	0.0020	0.0164	0.0409	0.0006	0.0262	0.3275	0.0079	0.1203
**HDL, mmol/l**								
Baseline	1.14 (0.01)	1.27 (0.02)	1.08 (0.01)	1.26 (0.01)	1.13 (0.01)	1.20 (0.04)	1.22 (0.01)	1.11 (0.03)
End-point	1.15 (0.01)	1.25 (0.02)	1.09 (0.01)	1.26 (0.01)	1.15 (0.01)	1.19 (0.04)	1.22 (0.01)	1.12 (0.02)
Δ from baseline	0.02 (0.01, 0.02)	−0.03 (−0.06, −0.01)	0.02 (0.01, 0.03)	−0.00 (−0.02, 0.01)	0.01 (0.00, 0.02)	−0.00 (−0.05, 0.04)	0.00 (−0.01, 0.02)	0.01 (−0.02, 0.04)
p Value*	0.0005	0.0173	0.0014	0.6923	0.0451	0.8507	0.6298	0.5385
**LDL, mmol/l**								
Baseline	2.66 (0.03)	2.55 (0.05)	2.54 (0.03)	2.75 (0.04)	2.62 (0.03)	2.75 (0.13)	2.59 (0.04)	2.88 (0.08)
End-point	2.57 (0.02)	2.37 (0.05)	2.45 (0.03)	2.65 (0.03)	2.52 (0.03)	2.67 (0.13)	2.49 (0.04)	2.76 (0.08)
Δ from baseline	−0.08 (−0.11, −0.04)	−0.17 (−0.25, −0.09)	−0.09 (−0.13, −0.04)	−0.10 (−0.15, −0.05)	−0.10 (−0.15, −0.06)	−0.08 (−0.28, 0.12)	−0.09 (−0.15, −0.03)	−0.09 (−0.19, 0.01)
p Value*	< 0.0001	< 0.0001	0.0001	0.0001	< 0.0001	0.4290	0.0038	0.0712
**Non-HDL, mmol/l**								
Baseline	3.57 (0.03)	3.32 (0.06)	3.45 (0.03)	3.62 (0.04)	3.57 (0.04)	3.48 (0.16)	3.39 (0.04)	3.72 (0.09)
End-point	3.42 (0.03)	3.12 (0.05)	3.29 (0.03)	3.46 (0.04)	3.39 (0.03)	3.41 (0.15)	3.24 (0.04)	3.55 (0.09)
Δ from baseline	−0.14 (−0.19, −0.10)	−0.20 (−0.28, −0.11)	−0.15 (−0.20, −0.10)	−0.16 (−0.22, −0.09)	−0.17 (−0.22, −0.12)	−0.09 (−0.32, 0.14)	−0.14 (−0.20, −0.07)	−0.15 (−0.26, −0.03)
p Value*	< 0.0001	< 0.0001	< 0.0001	< 0.0001	< 0.0001	0.4251	< 0.0001	0.0126
**Total cholesterol, mmol/l**								
Baseline	4.71 (0.03)	4.59 (0.07)	4.52 (0.03)	4.89 (0.04)	4.70 (0.04)	4.68 (0.16)	4.61 (0.04)	4.83 (0.09)
End-point	4.57 (0.03)	4.36 (0.06)	4.38 (0.03)	4.72 (0.04)	4.54 (0.04)	4.60 (0.15)	4.47 (0.04)	4.66 (0.09)
Δ from baseline	−0.13 (−0.17, −0.08)	−0.23 (−0.32, −0.13)	−0.13 (−0.18, −0.08)	−0.16 (−0.22, −0.10)	−0.16 (−0.21, −0.10)	−0.10 (−0.34, 0.14)	−0.13 (−0.20, −0.06)	−0.14 (−0.25, −0.02)
p Value*	< 0.0001	< 0.0001	< 0.0001	< 0.0001	< 0.0001	0.4204	0.0002	0.0212
**Triglycerides, mmol/l**								
Baseline	2.14 (0.05)	1.76 (0.06)	2.14 (0.05)	2.00 (0.05)	2.21 (0.06)	1.73 (0.24)	1.86 (0.06)	2.02 (0.11)
End-point	1.96 (0.04)	1.71 (0.06)	1.97 (0.05)	1.86 (0.04)	2.02 (0.05)	1.83 (0.22)	1.73 (0.04)	1.89 (0.07)
Δ from baseline	−0.18 (−0.26, −0.09)	−0.05 (−0.14, 0.04)	−0.17 (−0.27, −0.06)	−0.14 (−0.23, −0.05)	−0.19 (−0.29, −0.08)	0.03 (−0.28, 0.35)	−0.12 (−0.22, −0.02)	−0.13 (−0.32, 0.06)
p Value*	< 0.0001	0.3049	0.0022	0.0020	0.0005	0.8366	0.0167	0.1750

FG, fasting glucose; SBP, systolic blood pressure; DBP, diastolic blood pressure; HDL, high-density lipoprotein cholesterol; LDL, low-density lipoprotein cholesterol; Chol, cholesterol. Unless otherwise noted, all baseline and end-point values are mean

(SE) and all Δ from baseline values are mean

(95% confidence interval).

* Paired *t*-test for end-point compared with baseline.

#### Gender

Significant improvements from baseline in A1C, FG, body weight, blood pressure and lipids were seen in both male and female patients in response to EQW (Table 3; Figure 2).

#### Race

Significant improvements from baseline in A1C, FG and body weight were evident in all of the racial subpopulations treated with EQW (Table 3; Figure 2). Significant improvements in both systolic and diastolic blood pressure were seen in the White and Asian subpopulations, a significant improvement in systolic blood pressure was seen in the Hispanic subpopulation and no significant change in blood pressure was seen in the Black subpopulation (Table 3). Significant improvements in all of the lipid parameters were seen in the White and Asian subpopulations [other than high-density lipoprotein (HDL) cholesterol in the Asian subpopulation], significant improvements in non-HDL cholesterol and total cholesterol were seen in the Hispanic subpopulation and no significant change in lipid parameters were seen in the Black subpopulation (Table 3).

#### Duration of diabetes

Significant improvements from baseline in A1C, FG, body weight and lipids (except HDL cholesterol) were seen in patients with a shorter (< 10 years) or longer (≥ 10 years) duration of diabetes (Table 4; Figure 2). Significant improvements in both systolic and diastolic blood pressure from baseline were seen in patients with a shorter duration of diabetes, whereas a significant improvement only in systolic blood pressure was seen in patients with a longer duration of diabetes (Table 4).

**Table 4:** Efficacy end-points in the duration of diabetes and body mass index subpopulations

Parameter	Duration of diabetes		Body mass index (kg/m^2^)				
	
	< 10 years (*N* = 1268)	≥ 10 years (*N* = 451)	< 25 (*N* = 216)	≥ 25 to < 30 (*N* = 560)	≥ 30 to < 35 (*N* = 512)	≥ 35 to < 40 (*N* = 266)	≥ 40 (*N* = 154)
**A1C, %**							
Baseline	8.4 (0.03)	8.6 (0.05)	8.7 (0.07)	8.5 (0.05)	8.4 (0.05)	8.4 (0.07)	8.5 (0.09)
End-point	7.0 (0.03)	7.2 (0.05)	7.2 (0.08)	7.0 (0.05)	7.1 (0.05)	7.1 (0.08)	7.1 (0.09)
Δ from baseline	−1.4 (−1.5, −1.3)	−1.4 (−1.5, −1.3)	−1.5 (−1.7, −1.4)	−1.5 (−1.6, −1.4)	−1.3 (−1.4, −1.2)	−1.4 (−1.5, −1.2)	−1.5 (−1.7, −1.3)
p Value*	< 0.0001	< 0.0001	< 0.0001	< 0.0001	< 0.0001	< 0.0001	< 0.0001
**FG, mmol/l**							
Baseline	9.38 (0.07)	9.81 (0.13)	9.29 (0.18)	9.32 (0.11)	9.56 (0.11)	9.70 (0.18)	9.88 (0.21)
End-point	7.47 (0.07)	7.62 (0.12)	7.16 (0.17)	7.24 (0.10)	7.71 (0.10)	7.80 (0.17)	7.82 (0.20)
Δ from baseline	−1.90 (−2.05, −1.76)	−2.16 (−2.44, −1.88)	−2.11 (−2.51, −1.70)	−2.06 (−2.28, −1.85)	−1.81 (−2.03, −1.60)	−1.95 (−2.36, −1.54)	−2.13 (−2.56, −1.70)
p Value*	< 0.0001	< 0.0001	< 0.0001	< 0.0001	< 0.0001	< 0.0001	< 0.0001
**Weight, kg**							
Baseline	88.9 (0.57)	83.3 (0.96)	62.3 (0.56)	76.1 (0.46)	91.5 (0.55)	105.2 (0.87)	120.5 (1.20)
End-point	86.7 (0.57)	80.5 (0.93)	61.0 (0.57)	74.2 (0.48)	89.3 (0.58)	102.2 (0.93)	116.0 (1.25)
Δ from baseline	−2.2 (−2.4, −2.0)	−2.7 (−3.0, −2.3)	−1.4 (−1.8, −1.1)	−1.9 (−2.1, −1.6)	−2.2 (−2.5, −1.9)	−3.2 (−3.7, −2.6)	−4.5 (−5.4, −3.7)
p Value*	< 0.0001	< 0.0001	< 0.0001	< 0.0001	< 0.0001	< 0.0001	< 0.0001
**SBP, mmHg**							
Baseline	130 (0.4)	132 (0.8)	127 (1.1)	130 (0.6)	132 (0.6)	131 (0.9)	133 (1.2)
End-point	127 (0.4)	129 (0.8)	123 (1.1)	127 (0.6)	129 (0.6)	129 (0.9)	130 (1.3)
Δ from baseline	−2.9 (−3.7, −2.2)	−3.6 (−5.1 −2.1)	−4.2 (−6.3, −2.1)	−3.2 (−4.4, −2.0)	−3.1 (−4.3, −1.9)	−2.3 (−3.8, −0.8)	−3.0 (−5.2, −0.8)
p Value*	< 0.0001	< 0.0001	0.0001	< 0.0001	< 0.0001	0.0035	0.0087
**DBP, mmHg**							
Baseline	80 (0.2)	77 (0.4)	76 (0.6)	78 (0.4)	80 (0.4)	80 (0.6)	81 (0.8)
End-point	79 (0.3)	77 (0.4)	75 (0.6)	78 (0.4)	79 (0.4)	80 (0.6)	80 (0.8)
Δ from baseline	−1.1 (−1.6, −0.6)	−0.1 (−0.9, 0.7)	−1.2 (−2.5, 0.1)	−0.4 (−1.2, 0.3)	−1.5 (−2.2, −0.8)	−0.0 (−1.1, 1.0)	−1.4 (−3.1, 0.3)
p Value*	< 0.0001	0.8007	0.0681	0.2638	< 0.0001	0.9858	0.1105
**HDL, mmol/l**							
Baseline	1.14 (0.01)	1.22 (0.02)	1.33 (0.03)	1.18 (0.01)	1.11 (0.01)	1.11 (0.02)	1.11 (0.02)
End-point	1.15 (0.01)	1.23 (0.02)	1.31 (0.02)	1.18 (0.01)	1.13 (0.01)	1.12 (0.02)	1.14 (0.02)
Δ from baseline	0.01 (−0.00, 0.02)	0.01 (−0.01, 0.02)	−0.01 (−0.04, 0.01)	−0.00 (−0.02, 0.01)	0.02 (0.00, 0.03)	0.01 (−0.01, 0.03)	0.03 (0.00, 0.06)
p Value*	0.0700	0.5650	0.3450	0.8421	0.0217	0.1677	0.0255
**LDL, mmol/l**							
Baseline	2.66 (0.03)	2.56 (0.04)	2.69 (0.06)	2.67 (0.04)	2.62 (0.04)	2.63 (0.07)	2.56 (0.07)
End-point	2.59 (0.03)	2.40 (0.04)	2.55 (0.06)	2.53 (0.04)	2.51 (0.04)	2.60 (0.06)	2.52 (0.07)
Δ from baseline	−0.07 (−0.11, −0.03)	−0.15 (−0.21, −0.09)	−0.12 (−0.21, −0.03)	−0.13 (−0.19, −0.07)	−0.11 (−0.17, −0.04)	0.00 (−0.08, 0.09)	−0.06 (−0.16, 0.05)
p Value*	0.0004	< 0.0001	0.0067	< 0.0001	0.0012	0.9865	0.2778
**Non-HDL, mmol/l**							
Baseline	3.58 (0.03)	3.37 (0.05)	3.44 (0.07)	3.51 (0.05)	3.57 (0.05)	3.57 (0.08)	3.52 (0.08)
End-point	3.44 (0.03)	3.15 (0.05)	3.27 (0.07)	3.33 (0.04)	3.42 (0.05)	3.45 (0.07)	3.36 (0.08)
Δ from baseline	−0.13 (−0.18, −0.09)	−0.21 (−0.27, −0.14)	−0.16 (−0.26, −0.06)	−0.18 (−0.25, −0.11)	−0.14 (−0.22, −0.07)	−0.10 (−0.20, −0.00)	−0.18 (−0.30, −0.06)
p Value*	< 0.0001	< 0.0001	0.0016	< 0.0001	0.0002	0.0488	0.0035
**Total cholesterol, mmol/l**							
Baseline	4.72 (0.03)	4.60 (0.05)	4.77 (0.07)	4.69 (0.05)	4.68 (0.05)	4.68 (0.08)	4.63 (0.08)
End-point	4.59 (0.03)	4.38 (0.05)	4.58 (0.07)	4.51 (0.04)	4.55 (0.05)	4.57 (0.07)	4.49 (0.08)
Δ from baseline	−0.12 (−0.17, −0.07)	−0.20 (−0.27, −0.13)	−0.18 (−0.28, −0.07)	−0.18 (−0.25, −0.11)	−0.13 (−0.20, −0.05)	−0.09 (−0.19, 0.02)	−0.15 (−0.28, −0.02)
p Value*	< 0.0001	< 0.0001	0.0008	< 0.0001	0.0014	0.1025	0.0232
**Triglycerides, mmol/l**							
Baseline	2.16 (0.05)	1.86 (0.06)	1.73 (0.11)	1.97 (0.06)	2.22 (0.07)	2.24 (0.14)	2.25 (0.11)
End-point	2.00 (0.04)	1.70 (0.05)	1.61 (0.07)	1.87 (0.05)	2.07 (0.07)	1.96 (0.07)	1.96 (0.08)
Δ from baseline	−0.16 (−0.24, −0.07)	−0.15 (−0.24, −0.06)	−0.12 (−0.31, 0.08)	−0.09 (−0.18, 0.00)	−0.14 (−0.30, 0.02)	−0.30 (−0.50, −0.09)	−0.29 (−0.44, −0.13)
p Value*	0.0007	0.0013	0.2273	0.0535	0.0888	0.0057	0.0003

FG, fasting glucose; SBP, systolic blood pressure; DBP, diastolic blood pressure; HDL, high-density lipoprotein cholesterol; LDL, low-density lipoprotein cholesterol; Chol, cholesterol. Unless otherwise noted, all baseline and end-point values are mean (SE) and all Δ from baseline values are mean (95% confidence interval).

* Paired *t*-test for end-point compared with baseline.

#### Body mass index

Significant improvements from baseline in A1C, FG, body weight and systolic blood pressure were evident in all of the BMI subpopulations (Table 4; Figure 2). A significant improvement in diastolic blood pressure from baseline was evident in the ≥ 30- to < 35-kg/m^2^ subpopulation only (Table 4). Significant improvements from baseline in low-density lipoprotein (LDL) cholesterol, non-HDL and total cholesterol were evident in the < 25 kg/m^2^ and ≥ 25 to < 30 kg/m^2^ subpopulations (Table 4). Significant improvements from baseline in HDL, LDL, non-HDL and total cholesterol were evident in the ≥ 30- to < 35-kg/m^2^ subpopulation (Table 4). Significant improvements from baseline in non-HDL and triglycerides were evident in the ≥ 35- to < 40-kg/m^2^ subpopulation (Table 4). Significant improvements from baseline in HDL, non-HDL, total cholesterol and triglycerides were evident in the ≥ 40-kg/m^2^ subpopulation (Table 4).

### Tolerability

Overall, the most common AEs were hypoglycaemia (16.4%), nausea (14.7%), diarrhoea (10.9%) and nasopharyngitis (7.2%) (see Tables 5 and 6 for common AEs by subpopulation). Hypoglycaemia was more common among patients who were taking a concomitant SU than that among patients who were not (Tables 5 and 6), and there were no episodes of major hypoglycaemia.

**Table 5:** Tolerability end-points in the age, gender and racial subpopulations

Parameter, no. (%)	Age		Gender		Race			
		
	< 65 years (*N* = 1427)	≥ 65 years (*N* = 292)	Male (*N* = 944)	Female (*N* = 775)	White (*N* = 1000)	Black (*N* = 47)	Asian (*N* = 505)	Hispanic (*N* = 147)
Nausea	219 (15.3)	33 (11.3)	95 (10.1)	157 (20.3)	159 (15.9)	9 (19.1)	61 (12.1)	22 (15.0)
Diarrhoea	151 (10.6)	36 (12.3)	87 (9.2)	100 (12.9)	105 (10.5)	10 (21.3)	55 (10.9)	15 (10.2)
Nasopharyngitis	103 (7.2)	20 (6.8)	69 (7.3)	54 (7.0)	82 (8.2)	1 (2.1)	28 (5.5)	12 (8.2)
Headache	102 (7.1)	18 (6.2)	46 (4.9)	74 (9.5)	84 (8.4)	8 (17.0)	15 (3.0)	11 (7.5)
Injection-site nodule	109 (7.6)	9 (3.1)	62 (6.6)	56 (7.2)	77 (7.7)	2 (4.3)	34 (6.7)	4 (2.7)
Vomiting	97 (6.8)	15 (5.1)	40 (4.2)	72 (9.3)	61 (6.1)	5 (10.6)	41 (8.1)	5 (3.4)
Constipation	78 (5.5)	21 (7.2)	47 (5.0)	52 (6.7)	57 (5.7)	2 (4.3)	32 (6.3)	6 (4.1)
Injection-site pruritus	76 (5.3)	13 (4.5)	40 (4.2)	49 (6.3)	45 (4.5)	5 (10.6)	29 (5.7)	9 (6.1)
AELW	58 (4.1)	14 (4.8)	34 (3.6)	38 (4.9)	46 (4.6)	1 (2.1)	18 (3.6)	6 (4.1)
Hypoglycaemia								
With an SU*	75/590 (12.7)	18/150 (12.0)	47/414 (11.4)	46/326 (14.1)	56/389 (14.4)	0/9 (0.0)	29/311 (9.3)	8/27 (29.6)
Without an SU*	17/837 (2.0)	6/142 (4.2)	13/530 (2.5)	10/449 (2.2)	17/611 (2.8)	0/38 (0.0)	5/194 (2.6)	1/120 (0.8)

AELW, adverse event leading to withdrawal; SU, sulfonylurea.

* *n*/*N* (%).

**Table 6:** Tolerability end-points in the duration of diabetes and body mass index subpopulations

Parameter, no. (%)	Duration of diabetes		Body mass index (kg/m^2^)				
	
	< 10 years (*N* = 1268)	≥ 10 years (*N* = 451)	< 25 (*N* = 216)	≥ 25 to < 30 (*N* = 560)	≥ 30 to < 35 (*N* = 512)	≥ 35 to < 40 (*N* = 266)	≥ 40 (*N* = 154)
Nausea	178 (14.0)	74 (16.4)	27 (12.5)	73 (13.0)	80 (15.6)	44 (16.5)	27 (17.5)
Diarrhoea	137 (10.8)	50 (11.1)	19 (8.8)	60 (10.7)	51 (10.0)	33 (12.4)	24 (15.6)
Nasopharyngitis	89 (7.0)	34 (7.5)	15 (6.9)	41 (7.3)	43 (8.4)	12 (4.5)	12 (7.8)
Headache	87 (6.9)	33 (7.3)	10 (4.6)	35 (6.3)	33 (6.4)	25 (9.4)	17 (11.0)
Injection-site nodule	98 (7.7)	20 (4.4)	17 (7.9)	35 (6.3)	40 (7.8)	17 (6.4)	9 (5.8)
Vomiting	79 (6.2)	33 (7.3)	19 (8.8)	39 (7.0)	27 (5.3)	18 (6.8)	9 (5.8)
Constipation	78 (6.2)	21 (4.7)	13 (6.0)	34 (6.1)	32 (6.3)	17 (6.4)	2 (1.3)
Injection-site pruritus	67 (5.3)	22 (4.9)	10 (4.6)	23 (4.1)	31 (6.1)	14 (5.3)	10 (6.5)
AELW	55 (4.3)	17 (3.8)	9 (4.2)	33 (5.9)	18 (3.5)	7 (2.6)	5 (3.2)
Hypoglycaemia							
With an SU*	54/456 (11.8)	39/284 (13.7)	11/137 (8.0)	41/247 (16.6)	22/203 (10.8)	12/86 (14.0)	6/58 (10.3)
Without an SU*	18/812 (2.2)	5/167 (3.0)	3/79 (3.8)	9/313 (2.9)	4/309 (1.3)	5/180 (2.8)	2/96 (2.1)

AELW, adverse event leading to withdrawal; SU, sulfonylurea.

* *n*/*N* (%).

The rates of withdrawals because of AEs were low across all subpopulations (range, 2.1–5.9%); the most common AEs leading to withdrawal overall were injection-site pruritus (*n* = 8, 0.5%), nausea (*n* = 6, 0.3%) and diarrhoea (*n* = 5, 0.3%).

## Discussion

Considering that nearly 26 million people in the USA have diabetes (17), the demographic and physiological characteristics of individual patients within that population vary widely. Typical clinical studies for diabetes drug development are powered to assess glycaemic control against placebo or active comparators for the overall population and often lack sufficient power to assess drug effects within subpopulations of patients. Use of aggregate data from similarly designed trials can increase patient numbers to allow for exploratory analysis of more specifically defined populations.

In these pooled analyses of seven phase 3 EQW studies, clinical data were stratified by baseline age, gender, race, duration of diabetes and BMI. Results of these analyses were consistent with those of the individual trials and showed that the treatment with EQW was associated with significant improvements in glycaemic control from baseline (i.e. A1C and FG) across these different baseline characteristics. Results also showed that significant improvements in body weight were observed in all of the subpopulations. No across-subpopulation statistical comparisons were conducted; however, results of these analyses may be used to qualitatively compare efficacy and tolerability variables across different subpopulations of patients.

Changes in A1C and FG from baseline were fairly similar across subpopulations. Although body weight was significantly reduced in all subpopulations, there was a tendency for greater improvement in the female subpopulation than in the male subpopulation. In addition, there appeared to be progressively greater weight loss with higher baseline BMI.

The use of EQW was associated with significant improvements in systolic blood pressure in all subpopulations other than the Black subpopulation. Significant improvements in diastolic blood pressure were observed in all of the age and gender subpopulations. Diastolic blood pressure also significantly improved in the White, Asian, duration of diabetes < 10 years and BMI ≥ 30 to < 35 kg/m^2^ subpopulations. Overall, significant improvements in lipid parameters were generally observed in all of the age (other than triglycerides in the ≥ 65-year subpopulation), gender (other than HDL cholesterol in the female subpopulation) and duration of diabetes (other than HDL cholesterol) subpopulations. Significant changes in lipids were scattered in the BMI subpopulations. In general, significant improvements in lipid parameters were seen in the White and Asian (other than HDL cholesterol) subpopulations but not in the Black or Hispanic (other than non-HDL and total cholesterol) subpopulations. Together, these data show that many of the subpopulations had significant improvements in blood pressure and/or lipid parameters other than the Black subpopulation.

Evaluation of the tolerability data showed that the most common AEs overall were gastrointestinal in nature (i.e. nausea and diarrhoea). There did not appear to be any striking differences in the incidences of AEs between subpopulations other than a higher incidence of nausea, vomiting and diarrhoea in females than in males. The reason for this gender difference remains unknown and warrants further investigation. Of note, nausea is typically mild to moderate in intensity and the incidence of nausea decreases over time with continued EQW treatment (14). Injection-site nodules were a commonly reported AE. A report on the EQW formulation suggested that injection-site nodules were the result of a mild foreign body reaction in response to the microspheres of the EQW formulation (2). It was also noted that nodules were typically transient and generally resolved without medical intervention (2). Consistent with the individual clinical trials, hypoglycaemia was primarily associated with concomitant SU use. This safety profile of is consistent with that of a similar pooled analysis of exenatide BID studies (8,9), with the exception of a higher incidence of injection-site reactions with EQW. Furthermore, studies that compared EQW with exenatide BID noted a lower incidence of nausea and vomiting associated with EQW (10,14).

A strength of these analyses was that individual patient data for each of the trials was used, in contrast with typical meta-analyses that use aggregate summary statistics for individual trials. Moreover, the designs of the phase 3 studies for EQW included herein were similar enough to allow for pooled analyses. Limitations of the current analyses include the small numbers of patients in some subpopulations, the lack of a control arm and no adjustment for potentially confounding variables.

In conclusion, the results of these pooled analyses revealed that the treatment with EQW was associated with significant improvements in glycaemic control and body weight, irrespective of age, gender, race, duration of diabetes or BMI. Other than hypoglycaemia, the most common AEs overall were gastrointestinal in nature.
